
JOURNAL INFORMATION
==============================
NLM Title Abbreviation: Wirtschaftsdienst
Journal ID: Wirtschaftsdienst
NLM Journal ID: 101086612

Wirtschaftsdienst (Hamburg, Germany : 1949)

ISSN: 0043-6275

ARTICLE INFORMATION
==============================
PMCID: PMC7964514
PMID: 33746302
DOI: 10.1007/s10273-021-2869-6
Article ID: 2869
Article version: 1
Subjects: Zeitgespräch

Öffentliche Investitionen im Konjunkturprogramm als Einstieg in die sozial-ökologische Transformation

Dullien Sebastian Sebastian-Dullien@boeckler.de
1
Rietzler Katja Katja-Rietzler@BOECKLER.DE
2
Tober Silke silke-tober@boeckler.de
2
1 Institut für Makroökonomie u. Konjunkturforschung, Hans-Böckler-Straße 39, 40476 Düsseldorf, Deutschland
2 Ref. Geldpolitik, Institut für Makroökonomie u. Konjunkturforschung, Hans-Böckler-Straße 39, 40476 Düsseldorf, Deutschland
Electronic publication date: 2021 Jun 23
Print publication date: 2021
Volume: 101
Issue: 3
First page: 172
Last page: 175
Copyright: © Der/die Autor:in(nen) 2021
License: Open Access: Dieser Artikel wird unter der Creative Commons Namensnennung 4.0 International Lizenz veröffentlicht (https://creativecommons.org/licenses/by/4.0/deed.de).
Open Access wird durch die ZBW — Leibniz-Informationszentrum Wirtschaft gefördert.
License URL: https://creativecommons.org/licenses/by/4.0/

issue-copyright-statement: © The Author(s) 2021

==============================
Prof. Dr. Sebastian Dullien lehrt Volkswirtschaftslehre an der Hochschule für Technik und Wirtschaft Berlin und ist wissenschaftlicher Direktor des Instituts für Makroökonomie und Konjunkturforschung der Hans-Böckler-Stiftung (IMK) in Düsseldorf.

In den vergangenen Jahren hat sich in Deutschland zunehmend die Überzeugung durchgesetzt, dass die öffentliche Hand zu wenig investiert – in traditionelle Infrastruktur, aber auch in Zukunftsaufgaben wie die Dekarbonisierung, die Bildung oder den Ausbau der Kinderbetreuung. In einer Gemeinschaftsarbeit schätzten das Institut für Makroökonomie und Konjunkturforschung (IMK) gemeinsam mit dem Institut der deutschen Wirtschaft (IW) den Bedarf an öffentlichen Investitionen in Deutschland für die kommenden zehn Jahre auf 457 Mrd. Euro in Preisen von 2019 (Bardt et al., 2019). Nach einer längeren Debatte kam letztlich auch der wissenschaftliche Beirat beim Bundesministerium für Wirtschaft und Energie zu dem Schluss, dass Investitionsbedarfe in dieser Größenordnung „nicht unplausibel“ seien (Wissenschaftlicher Beirat beim Bundesministerium für Wirtschaft und Energie, 2020).

Seit Veröffentlichung der IMK-IW-Studie sind zusätzliche Investitionsbedarfe deutlich geworden, die in der ursprünglichen Schätzung nicht abgedeckt waren. So hat die EU Ende 2020 ihre Klimaziele verschärft. Statt einer Reduktion der Treibhausgasemissionen bis 2030 gegenüber 1990 um 40 % wird jetzt eine Reduktion um mindestens 55 % als Ziel angestrebt. Eine solche Beschleunigung des Strukturwandels bedeutet größere Investitionsbedarfe insbesondere in der ersten Hälfte der 2020er Jahre, und damit auch größere Investitionsanforderungen an die öffentliche Hand.

Ebenfalls in der Schätzung bislang explizit ausgeklammert waren Investitionen im Gesundheitssektor. Die COVID-19-Pandemie hat in den Gesundheitsämtern große Investitionsbedarfe schmerzhaft deutlich gemacht, wenn etwa Testergebnisse per Fax übermittelt wurden, weil keine moderne IT-Infrastruktur vorhanden war.

Gleichzeitig hat die wirtschaftspolitische Reaktion in der Corona-Krise dazu geführt, dass auch die Ausgaben für öffentliche Investitionen in die Höhe gefahren wurden. Insbesondere in der Zukunftskomponente des im Juni 2020 von der Bundesregierung verabschiedeten „Konjunktur- und Zukunftspakets“ mit einem Volumen von rund 170 Mrd. Euro sind signifikante Mittel für öffentliche Investitionen enthalten (Dullien et al., 2020). Auf EU-Ebene soll das „NextGenerationEU“-Aufbauprogramm die öffentlichen Investitionen in allen Mitgliedstaaten ankurbeln.

## Stabilisierung und die Rolle von Investitionen

Von den späten 1980ern bis zur globalen Finanzkrise wurde der Geldpolitik die entscheidende Rolle bei der makroökonomischen Stabilisierung zugemessen, während aktive Fiskalpolitik als wirkungslos oder gar schädlich interpretiert wurde. Für die Geldpolitik sprachen ihre schnelle Umsetzung und Wirkung. Mit anhaltender Dauer der Zeit der „Great Moderation“ stieg auch der Respekt vor den Notenbankern und brachte dem Chef der international führenden Zentralbank, der US-amerikanischen Federal Reserve, Alan Greenspan, die Titel „Maestro“ und „Almighty Alan“ ein. Während der globalen Finanz- und Wirtschaftskrise 2008/2009 stießen die Zentralbanken dann aber an die bereits von Keynes benannten Grenzen: Zinssenkungen laufen ins Leere, wenn Unternehmen nicht investieren wollen und private Haushalte aus Vorsicht sparen, statt zu konsumieren.

Die Rückbesinnung auf die Fiskalpolitik ging einher mit dem Bestreben Konjunkturprogramme „timely, targeted and temporary“, also in ihrer Wirkung rechtzeitig, gezielt und zeitlich begrenzt, zu gestalten (Elmendorf und Furman, 2008). Atkinson (2008) fügte diesen drei T’s das vierte T für „transformative“ hinzu, das angesichts der drohenden Klimakrise zunehmend an Bedeutung gewinnt (Bach et al., 2020; Dullien et al., 2020).

In diesem Rahmen gibt es zahlreiche Möglichkeiten die Konjunktur anzukurbeln. So regen Konsumgutscheine für energiesparende Haushaltsgeräte zeitnah den privaten Konsum an und schonen zugleich das Klima. Eine zeitlich beschränkte Sofortabschreibung von Ausrüstungen, die die Energiebilanz des Unternehmens verbessern, stimuliert die Investitionen (Bach et al., 2020). Allerdings zeigt sich, dass steuerliche Anreize und Transfers einen geringeren Multiplikator haben als öffentliche Investitionen (Gechert und Rannenberg, 2018). Dabei sind öffentliche Investitionen in Krisenzeiten besonders wirksam und können Multiplikatoren von über 2 hervorbringen, also eine Steigerung des Bruttoinlandsprodukts (BIP), die doppelt so hoch ist wie die eingesetzten Mittel (IWF, 2020).

In makroökonomischen Standardmodellen steigen die Zinsen infolge einer expansiven Fiskalpolitik, sodass die höheren Finanzierungskosten private Investitionen verdrängen. Bleiben die Zinsen aber unverändert niedrig – was aktuell im Euroraum infolge der tiefen Krise und der seit Jahren zu niedrigen Inflation wahrscheinlich ist – wirken die Investitionen gänzlich als zusätzliche Nachfrage.

Öffentliche Investitionen haben gegenüber anderen Fiskalmaßnahmen drei Vorteile: Sie erhöhen das Produktionspotenzial, regen private Investitionen an und können gezielt zur Förderung der Dekarbonisierung eingesetzt werden. Zwar haben öffentliche Investitionen gelegentlich einen längeren Planungsvorlauf als konsumfördernde Maßnahmen, die damit einhergehende Verringerung der Unsicherheiten über den künftigen wirtschaftspolitischen Kurs der Regierung fördert allerdings die private Investitionstätigkeit bereits unmittelbar. Für Investitionen, die zwangsläufig über mehrere Jahre angelegt sind, kann wegen der positiven Wirkung auf Erwartungen und Produktionspotenzial das Kriterium der zeitlichen Begrenzung entfallen.

## Transformative Elemente im Konjunkturpaket

Das Konjunkturelement im „Konjunktur- und Zukunftspaket“ der Bundesregierung mit einem ursprünglich angekündigten Volumen von 130 Mrd. Euro (3,8 % des Vorkrisen-BIP) erfüllt die Kriterien „timely“ und „temporary“, hätte aber hinsichtlich der Kriterien „targeted“ und „transformativ“ besser ausgestaltet werden können. Die größte Einzelmaßnahme – die auf sechs Monate begrenzte Senkung der Umsatzsteuersätze auf 16 % bzw. 5 % – ist mit 20 Mrd. Euro zugleich die am wenigsten zielgerichtete und transformative. Sie dürfte eine relativ geringe Multiplikatorwirkung gehabt haben, da sie nicht vollständig durch Preissenkungen weitergegeben wurde, auch Einkommensschichten mit hoher Sparquote zugutekam und die Sparquote, die sich im zweiten Quartal 2020 verdoppelt hatte, weiterhin hoch war. Eine klimapolitische Wirkung konnte sie nicht erzielen, weil sie nicht auf bestimmte Produkte beschränkt war. Ein Gegenbeispiel ist der Kinderbonus, der im Konjunkturpaket allerdings mit nur 4,3 Mrd. Euro gering bemessen war. Da nur Haushalte den Kinderbonus erhielten, die Kindergeld empfangen, nicht aber jene, die den Kinderfreibetrag nutzen, dürfte ein geringerer Anteil gespart worden sein als im Falle der Mehrwertsteuer. Zudem verringert der Kinderbonus grundsätzlich die Einkommens- und Chancenungleichheit und fördert damit die soziale Transformation (Bach et al., 2020).

Neben konsumorientierten Maßnahmen enthält das Konjunkturpaket zahlreiche reine Stützungsmaßnahmen, insbesondere für Kommunen (14,9 Mrd. Euro) und Unternehmen (25 Mrd. Euro). Maßnahmen mit investivem Charakter befinden sich primär im gleichzeitig verabschiedeten Zukunftspaket, darunter 9 Mrd. Euro für die Entwicklung und Anwendung der Wasserstofftechnologie, insgesamt 16 Mrd. Euro für die Bereiche künstliche Intelligenz, Quantentechnologie und Digitalisierung sowie 2,5 Mrd. Euro für die Förderung der Ladeinfrastruktur für Fahrzeuge mit alternativen Antrieben. Da die Kommunen einen erheblichen Investitionsstau vor sich herschieben (KfW Bankengruppe, 2020) und vor der Corona-Krise gerade erst begonnen hatten, verstärkt zu investieren, tragen Maßnahmen zur Stützung der Kommunen dabei entscheidend zur Stabilisierung der öffentlichen Investitionstätigkeit bei.

Insgesamt können von den 2020 und 2021 wirksam werdenden Maßnahmen mit einem Volumen von rund 130 Mrd. Euro knapp ein Viertel als transformativ gefasst werden, darunter die Förderung der E-Mobilität, des ÖPNV und der Bahn, die Aufstockung der $CO_2$-Gebäudesanierung, Bildungsinvestitionen, die gezielt transformative Förderung von F&E sowie 11 Mrd. Euro zur Stabilisierung der EEG-Umlage 2021 und 2022.

Mit dem European Green Deal setzt auch die Europäische Kommission insbesondere über die mittelfristige Finanzplanung und den Aufbaufonds „NextGenerationEU“ (NGEU) stark transformative Akzente und ist bestrebt, die nationalen Investitionen in Richtung Digitalisierung, Elektrifizierung und die Förderung der Wasserstofftechnologie zu lenken. Dabei stellt der Aufbaufonds für die EU-Länder gänzlich öffentliche Mittel zur Verfügung (750 Mrd. Euro), während die Förderung im Rahmen des mehrjährigen Finanzrahmens (ca. 1.100 Mrd. Euro) größtenteils über die Aktivierung privater Mittel, insbesondere durch das Programm „InvestEU“, mobilisiert werden sollen.

## Zukunftspaket erweitern und verstetigen

Mit dem Konjunktur- und Zukunftspaket hat die Bundesregierung insgesamt zusätzliche investive Maßnahmen im Umfang von über 60 Mrd. Euro in Aussicht gestellt[1]. Die auf den ersten Blick hohe Summe relativiert sich, wenn man berücksichtigt, dass es um ein Programm für mehrere Jahre und teilweise über 2025 hinaus geht. Im Durchschnitt entspricht das weniger als 0,2 % des BIP jährlich. Gemessen an dem von IMK und IW ermittelten jährlichen Bedarf von mindestens 1,3 % des BIP (Bardt et al., 2019) decken die Investitionen im Konjunktur- und Zukunftspaket somit nur einen Bruchteil der erforderlichen Maßnahmen ab. Hinzu kommt, dass die Umsetzung des Pakets teilweise nur schleppend anläuft. Das gilt beispielsweise für die nationale Wasserstoffstrategie, den mit 7 Mrd. Euro größten Einzelposten des Zukunftspakets.[2] Anstelle der ursprünglich für 2021 veranschlagten 1,7 Mrd. Euro sind nach Abschluss der Beratungen lediglich 0,4 Mrd. Euro eingeplant worden (Deutscher Bundestag, 2020).

Aus der Aufbau- und Resilienzfazilität erhält Deutschland 2021 bis 2023 über 25 Mrd. Euro, die für sechs Schwerpunkte verwendet werden sollen:[3] Klimaschutz und Energiewende, Digitalisierung der Wirtschaft und Infrastruktur, Digitalisierung der Bildung, soziale Teilhabe, Stärkung eines pandemieresistenten Gesundheitswesens, moderne öffentliche Verwaltung und Abbau von Investitionshemmnissen (BMF, 2020). Das sind Bereiche, bei denen ein großer Handlungsbedarf besteht. Allerdings werden die Mittel aus der Aufbau- und Resilienzfazilität nicht genutzt, um das Zukunftspaket noch einmal deutlich aufzustocken, sondern sie werden im Wesentlichen dazu verwendet, bereits geplante Maßnahmen des Konjunktur- und Zukunftspakets zu finanzieren. Die jüngsten Maßnahmen im Rahmen der Krisenbekämpfung schließen die Lücke bei den öffentlichen Investitionen bei Weitem noch nicht. In den kommenden Jahren müssen die Investitionen in die Transformation und die Infrastruktur also weiter angehoben und dann auf einem hohen Niveau verstetigt werden.

## Industriepolitik, um Wertschöpfung in Deutschland und Europa zu halten

Dabei geht es nicht nur um die Modernisierung der Infrastruktur und die Einhaltung vorgegebener Klimaziele, sondern auch um die Basis für den Wohlstand von morgen. Eine Investitionsstrategie muss die Entwicklung zukünftiger Geschäftsmodelle unterstützen, damit in Deutschland auch in Zukunft hochwertige Produkte für den Weltmarkt produziert werden und gut bezahlte Arbeitsplätze erhalten und neu geschaffen werden können.

Daher sollten die Investitionsprogramme in eine nationale und europäische Industriepolitik eingebettet werden. Die Corona-Krise hat die Verletzlichkeit internationaler Lieferketten deutlich vor Augen geführt. Die Systemkonkurrenz mit China um die Technologieführerschaft ist ein weiteres Argument, wesentliche Produktionskapazitäten in Deutschland bzw. Europa zu halten bzw. neu aufzubauen (Dullien, 2021). Neben der Digitalisierung und der Weiterentwicklung künstlicher Intelligenz liegt ein Fokus auf der Dekarbonisierung der Industrie, die z.B. mit dem Aufbau von Wertschöpfungsketten der Wasserstoffproduktion auch ein Wachstums- und Beschäftigungspotenzial bietet (DIW Econ und Wuppertal Institut, 2020).

Da Investitionen in neue klimaneutrale Technologien mit einem hohen Eigenkapitalbedarf verbunden sind, könnte zusätzlich zur bestehenden Förderlandschaft, die überwiegend mit zinsverbilligten Krediten und Zuschüssen operiert, ein kreditfinanzierter staatlicher Transformationsfonds ein wichtiger Baustein einer umfassenden Modernisierungsstrategie sein (Dullien et al., 2021). Staatliche Beteiligungen spielen bisher eine untergeordnete Rolle, haben aber gegenüber privaten Modellen den Vorteil, „geduldiges Kapital“ bereitzustellen. Gleichzeitig ergeben sich für den Staat Möglichkeiten von erfolgreichen Geschäftsmodellen zu profitieren (Laplane und Mazzucato, 2020). Bei der institutionellen Ausgestaltung sollte auf schlanke transparente Strukturen gesetzt werden, wobei auf das Know-How der KfW, der Bundesbank, der Bundesministerien und der Finanzagentur zurückgegriffen werden kann.

## Fiskalregeln investitionsfreundlicher gestalten

Während staatliche Beteiligungen als sogenannte finanzielle Transaktionen sowohl bei der Schuldenbremse als auch bei den europäischen Fiskalregeln von den Schuldengrenzen ausgenommen sind, besteht für die übrigen Investitionsbedarfe die Gefahr, dass sie in den kommenden Jahren nur unzureichend umgesetzt werden. Insbesondere, falls, wie in der aktuellen mittelfristigen Finanzplanung des Bundes aktuell noch vorgesehen, schnell zu einer unreformierten Schuldenbremse zurückgekehrt wird. Obwohl die NGEU-Mittel schon verplant sind und die allgemeine Rücklage (Flüchtlingsrücklage) aufgelöst werden soll, wird im Finanzplan noch ein substanzieller Handlungsbedarf von insgesamt 42,5 Mrd. Euro ermittelt, um die Schuldenbremse ab 2022 einzuhalten (Bundesrat, 2020). Revisionen der

1 Wobei allerdings 5 Mrd. Euro auf eine Eigenkapitalerhöhung für die Bahn entfallen und vorgezogene Investitionen des Bundes teilweise auch Rüstungsprojekte enthalten.

2 Zu den im Inland für die Wasserstoffstrategie eingeplanten 7 Mrd. Euro kommen 2 Mrd. Euro an Ausgaben für außenwirtschaftliche Partnerschaften zur Wasserstoffversorgung, sodass in der Summe 9 Mrd. Euro in die Wasserstoffstrategie fließen.

3 23,6 Mrd. Euro in Preisen von 2018. Das entspricht 25,6 Mrd. Euro in jeweiligen Preisen.

Konjunkturkomponente könnten den „Handlungsbedarf“ sogar noch erhöhen. Ab 2026 käme es durch die dann verstärkt einsetzenden Tilgungsausgaben des Bundes zu einer weiteren Verringerung der Haushaltsspielräume. Damit ist genau in dem für die Transformation der deutschen Wirtschaft besonders kritischen Zeitraum bis 2030 ein restriktiver finanzpolitischer Kurs zu befürchten, der die Zukunftschancen Deutschlands unnötigerweise beschränken könnte. Sinnvoll wäre eine schrittweise Rückkehr zu reformierten Fiskalregeln, bei denen eine „golden Rule” basierend auf einer pragmatischen Definition des Investitionsbegriffs ein zentraler Bestandteil wäre (Truger, 2015). Selbst bei großenteils kreditfinanzierten öffentlichen Investitionen, dürfte die staatliche Schuldenstandsquote langfristig deutlich rückläufig sein.

## Schlussfolgerungen

Zusammenfassend kann man sagen, dass die mit dem Konjunktur- und Zukunftspaket auf den Weg gebrachten Investitionen, aber auch die im Klimapaket angelegten Ausgaben, ein guter erster Schritt in die sozial-ökologische Transformation sind. Je nach genauer Ausgestaltung bietet zudem das NGEU-Programm das Potenzial, die Mitgliedsstaaten der Europäische Union ebenfalls auf diesem Pfad entscheidend zu unterstützen.

Gleichzeitig aber dürften die Mittel nicht ausreichend sein. Trotz des Anstiegs der öffentlichen Investitionen in der COVID-19-Pandemie liegen die aktuellen Investitionsausgaben in Deutschland spürbar unter dem, was Bardt et al. (2019) als notwendig geschätzt haben. Infolge der Anhebung des europäischen Ziels für Reduktion der Treibhausgasemissionen bis 2030 müsste zudem auch das deutsche Emissionsziel ambitionierter ausfallen, was den Investitionsbedarf kurzfristig noch zusätzlich erhöhen dürfte. Eine weitere Aufstockung und Verstetigung der (dann höheren) Investitionen ist deshalb dringend geboten.

Für eine erfolgreiche sozial-ökologische Transformation reichen zudem mehr Investitionsmittel allein nicht aus. Eine stärkere Nutzung von Preissignalen wie der jüngst eingeführten $CO_2$-Steuer und Regulierungsmaßnahmen, beispielsweise zur Förderung der Kreislaufwirtschaft, ist erforderlich. Es müssen zudem neue industriepolitische Instrumente wie etwa ein Transformationsfonds genutzt werden, um private Investitionen zu mobilisieren und technologische Innovationen zu fördern. Gleichzeitig müssen die Verwaltungen mit verbesserten Verfahren und Strukturen, aber auch mit zusätzlichem Personal ertüchtigt werden, die Transformation von staatlicher Seite voranzutreiben.


Dr. Katja Rietzler ist Referatsleiterin für Steuer- und Finanzpolitik am IMK.

Dr. Silke Tober ist Referatsleiterin für Geldpolitik am IMK.


Literatur

Atkinson, R. D. (2008), Timely, Targeted, Temporary and Transformative: Crafting an Innovation-Based Economic Stimulus Package. The Information Foundation and Innovation Foundation, https://itif.org/files/TimelyTargetedTemporaryTransformative.pdf (2. März 2021).
Bach, S., H. Bär, K. Bohnenberger, S. Dullien, C. Kemfert, M. Rehm, K. Rietzler, M. Runkel, S. Schmalz, S. Tober und A. Truger (2020), Sozialökologisch ausgerichtete Konjunkturpolitik in und nach der Corona-Krise. Forschungsvorhaben im Auftrag des Bundesministeriums für Umwelt, Naturschutz und nukleare Sicherheit, IMK Study, 68.
Bardt, H., S. Dullien, M. Hüther und K. Rietzler (2019), Für eine solide Finanzpolitik. Investitionen ermöglichen!, IMK Report, 152.
BMF — Bundesministerium der Finanzen (2020), Deutscher Aufbau- und Resilienzplan, Entwurf.
Bundesrat (2020), Unterrichtung durch die Bundesregierung. Finanzplan des Bundes 2020 bis 2024, Bundesratsdrucksache, 517/20 vom 9. Oktober.
Deutscher Bundestag (2020), Beschlussempfehlung des Haushaltsausschusses (8. Ausschuss) zu dem Entwurf eines Gesetzes über die Feststellung des Bundeshaushaltsplans für das Haushaltsjahr 2021 (Haushaltsgesetz 2021) — Drucksache 19/22600 — hier: Einzelplan 60. Bundestagsdrucksache, 19/23323.
DIW Econ und Wuppertal Institut (2020), Bewertung der Vor- und Nachteile von Wasserstoffimporten im Vergleich zur heimischen Erzeugung, Studie für den Landesverband Erneuerbare Energien NRW (LEE-NRW).
Dullien, S. (2021): Nach der Corona-Krise: Die nächste Phase der (De-) Globalisierung und die Rolle der Industriepolitik, IMK Policy Brief, Nr. 100, Januar.
Dullien, S., K. Rietzler und S. Tober (2021), Ein Transformationsfonds für Deutschland. Gutachten, IMK Study, 71.
Dullien S Tober S Truger A Wege aus der Wirtschaftskrise: Der Spagat zwischen Wachstumsstabilisierung und sozial-ökologischer Transformation WSI-Mitteilungen 2020 73 6 403 410 10.5771/0342-300X-2020-6-403
Elmendorf, D. W. und J. Furman (2008), If, When, How: A Primer on Fiscal Stimulus, Brookings Institution, https://www.brookings.edu/wp-content/uploads/2016/06/0110_fiscal_stimulus_elmendorf_furman.pdf (2. März 2021).
Gechert S Rannenberg A Which fiscal multipliers are regime-dependent? A meta-regression analysis Journal of Economics Survey 2018 32 4 1160 1182 10.1111/joes.12241
IWF — Internationaler Währungsfonds (2020), Fiscal Monitor: Policies for the Recovery.
KfW Bankengruppe (2020), KfW-Kommunalpanel 2020.
Laplane, A. und M. Mazzucato (2020), Socializing the risks and rewards of public investments: Economic, policy, and legal issues, Research Policy, x/2, Dezember, 100008.
Truger, A. (2015), Implementing the Golden Rule for Public Investment in Europe. Safeguarding Public Investment and Supporting the Recovery, Materialien zu Wirtschaft und Gesellschaft, 138, Arbeiterkammer Wien.
Wissenschaftlicher Beirat beim Bundesministerium für Wirtschaft und Energie (2020), Öffentliche Infrastruktur in Deutschland: Probleme und Reformbedarf.
